# Cardiotoxicity of trastuzumab in patients with HER2-positive gastric cancer

**DOI:** 10.18632/oncotarget.18700

**Published:** 2017-06-27

**Authors:** Ji Soo Park, Jong-Chan Youn, Chi Young Shim, Geu-Ru Hong, Choong-Kun Lee, Jee Hyung Kim, Hyung Soon Park, Su Jin Heo, Seung Hoon Beom, Hyo Song Kim, Sun Young Rha, Hyun Cheol Chung, Seok-Min Kang, Minkyu Jung

**Affiliations:** ^1^ Cancer Prevention Center, Yonsei Cancer Center, Seoul, Republic of Korea; ^2^ Division of Cardiology, Dongtan Sacred Heart Hospital, Hallym University College of Medicine, Hwaseong, Republic of Korea; ^3^ Division of Cardiology, Severance Cardiovascular Hospital, Yonsei University College of Medicine, Seoul, Republic of Korea; ^4^ Divison of Medical Oncology, Yonsei Cancer Center, Yonsei University College of Medicine, Seoul, Republic of Korea

**Keywords:** trastuzumab, cardiotoxicity, incidence, HER2, gastric cancer

## Abstract

Trastuzumab-induced cardiotoxicity (TIC) is the primary adverse event that limits the use of trastuzumab in HER2-positive breast cancer patients. However, the incidence and risk factors of TIC in HER2-positive gastric cancer are not known. Therefore, we evaluated the incidence and predictive factors of TIC in gastric cancer patients treated with trastuzumab in clinical practice. We reviewed cardiac dysfunction in HER2-positive gastric cancer patients between December 2005 and April 2015 in a prospectively-collected database that included prospective clinical trials at Yonsei Cancer Center, Republic of Korea. TIC was defined as an absolute decline in left ventricular ejection fraction (LVEF) of at least 10 percentage points from the baseline to a value less than 55%, as identified by a multiple-gated acquisition scan or an echocardiogram. Among the 115 patients, 70 patients (60.9%) received trastuzumab combined with chemotherapy, and 45 patients (39.1%) received chemotherapy alone as a first-line therapy. Symptomatic heart failure was not observed in either group, but a significant asymptomatic drop in LVEF was noted in five (7.1%) of the trastuzumab combined-group patients and in one (2.2%) chemotherapy-only group patient [hazard ratio (HR), 3.47; 95% confidence interval (CI), 0.40–29.8; *P*=0.257]. TIC was observed more frequently in elderly patients than in younger patients (HR, per age in year, 1.16; 95% CI, 1.02–1.31; *P*=0.019). Similar to prior observations in breast cancer, TIC in gastric cancer patients is not frequent or reversible. However, the asymptomatic drop in LVEF should be monitored continually in HER2-positive gastric cancer patients treated with trastuzumab, especially in elderly patients.

## INTRODUCTION

HER2 (ErbB2 or HER-2/neu) belongs to the human epidermal growth factor receptor (HER) family and is significantly correlated with the proliferation, migration, and differentiation of many kinds of cancer cells through its involvement in the activation of the PI3K/Akt and Ras/Raf/MEK/MAPK pathways. The monoclonal antibody trastuzumab, which targets HER2, is a well-known HER2-directed therapeutic drug [1]. This agent demonstrated improved survival outcomes in cancer patients, especially in cases of breast cancer associated with the amplification of *HER2* and/or overexpression of HER2 at the messenger RNA or protein level [2-4].

Meanwhile, because preclinical trastuzumab studies suggest that ErbB2 plays an essential role in the developing embryonic heart and is important for maintaining cardiac function in the adult heart [5], there are considerable concerns regarding cardiotoxicity in early clinical trials [2]. Although the primary risk for trastuzumab-induced cardiomyopathy (TIC) is reportedly the combination with anthracycline, trastuzumab without anthracycline also causes a reversible drop in left ventricular ejection fraction (LVEF) in 3.04–10.4% of incidence [6-10].

In gastric cancer, the Trastuzumab for Gastric Cancer (ToGA) trial investigated the efficacy of trastuzumab in HER2-positive metastatic gastric cancer patients. Though a low incidence of cardiotoxicity was found in this study (5% in the trastuzumab plus chemotherapy group and 1% in the chemotherapy group) [11], detailed profiles regarding LVEF in the treatment groups were not reported. Due to the lack of clinical trials and knowledge, the incidence and characteristics of cardiotoxicity related to trastuzumab in gastric cancer patients have not been clearly shown.

In this study, we tried to evaluate the incidence, characteristics, and clinical factors that were potentially related to TIC in patients with gastric cancer.

## RESULTS

### Patients characteristics

Between January 2006 and April 2015, we collected clinicopathological data from 115 patients who were diagnosed with inoperable, locally advanced, recurrent, or metastatic HER2-positive gastric cancer patients, and from participants in trastuzumab clinical trials at the Yonsei Cancer Center [NCT01041404 (ToGA trial), NCT01736410, and NCT0140696]. Among the patients, 70 received chemotherapy in combination with trastuzumab as a first-line treatment, whereas 45 were treated with chemotherapy only. Table 1 provides the baseline characteristics of the patients. Eighty-six patients (74.8%) were male and the median age at diagnosis was 58 (range, 30–80). Most of the clinical features were similar between the two groups. Of the four patients with underlying cardiac disease, one patient had persistent, asymptomatic atrial fibrillation, and the other three patients had coronary arterial diseases that were treated by percutaneous coronary intervention.

**Table 1:** Baseline characteristics of each treatment group

Variable		CTX only		Trastuzumab + CTX		Total		*P*-value
Number of patients		45		70		115		
Gender								
	Male	34	75.6%	52	74.3%	86	74.8%	0.878
	Female	11	24.4%	18	25.7%	29	25.2%	
Age								
	Median age, years (range)	58 (37–72)		59 (30–80)		58 (30–80)		0.884*
BMI								
	Median, kg/m^2^ (range)	22.7 (16.7–35.0)		21.4 (13.8–32.5)		21.9 (13.8–35.0)		0.059*
Baseline LVEF								
	Median, % (range)	66 (57–75)		65 (57–75)		66 (57–75)		0.307*
Maximally decreased LVEF from baseline LVEF								
	Median, % (range)	6 (1–12)		7 (1–21)		6 (1–21)		0.279*
Total treatment cycles								
	Median, number (range)	8 (2–92)		9 (1–62)		8 (1–92)		0.827*
Hypertension								
	Yes	12	26.7%	18	25.7%	30	26.1%	0.910
	No	33	73.3%	52	74.3%	85	73.9%	
Underlying cardiac disease								
	Yes	1	2.2%	3	4.3%	4	3.5%	>0.99†
	No	44	97.8%	67	95.7%	111	96.5%	
Diabetes								
	Yes	5	11.1%	8	11.4%	13	11.3%	>0.99†
	No	40	88.9%	62	88.6%	102	88.7%	
Hyperlipidemia								
	Yes	0	0.0%	2	2.8%	2	1.7%	0.519†
	No	45	100%	68	97.2%	113	98.3%	
Cerebrovascular disease								
	Yes	1	2.2%	3	4.3%	4	3.5%	>0.99†
	No	44	97.8%	67	95.7%	111	96.5%	
Smoking								
	Current	15	33.3%	20	28.6%	35	30.4%	0.862
	Former	11	24.4%	18	25.7%	29	25.2%	
	Never	19	42.3%	32	45.7%	51	44.4%	

BMI, body mass index; CTX, cytotoxic chemotherapy; LVEF, left ventricular ejection fraction; *Analyzed by the Mann-Whitney U test; † Analyzed by the Fisher’s exact test

### Treatment and changes in LVEF

The median baseline LVEF of all the patients was 66% (range, 57–75), and the median number of times LVEF measurements conducted was 4 (range, 2–21). For all patients included in this study, the median interval of cardiac monitoring was 12.0 weeks (range, 3.3–27.4). The median trastuzumab dose administered to the 70 patients who received both the drug and chemotherapy was 56 mg/kg (range, 8-374) over a median number of 9 cycles (range, 1–62) and a median of 6.4 months (range, 0.7–47.3). The 45 patients who were treated with chemotherapy-only received a median of 8 cycles (range, 2–92) of chemotherapy over 5.6 months (range, 0.5–65.2). The median follow-up duration was 58.2 months (95% CI, 49.6–67.2). As of November 30, 2015, 69 out of 115 patients (60.0%) experienced a drop in LVEF. The median percentage points of the decrease in LVEF from baseline was 6 (range, 1–19). Among the patients who experienced a drop in LVEF, six patients (five in the trastuzumab group, one in the chemotherapy only group) experienced a significant asymptomatic drop in LVEF of a median 11.5 percentage points (range, 10–19) from baseline LVEF.

### Cardiac dysfunction in the patients according to treatment with trastuzumab

In the patients who were treated with trastuzumab, 62.9% (44/70) experienced some degree of decline in LVEF, but the decreases were not significantly higher compared with patients treated with chemotherapy only [55.6% (25/45); *P*=0.435]. The median time to reach to maximal decreases in LVEF from the baseline LVEF measurement was 5.3 months (range, 1.4–30.4). However, no symptomatic congestive heart failure (CHF) or cardiac deaths occurred in the patients treated with or without trastuzumab.

**Figure 1:**
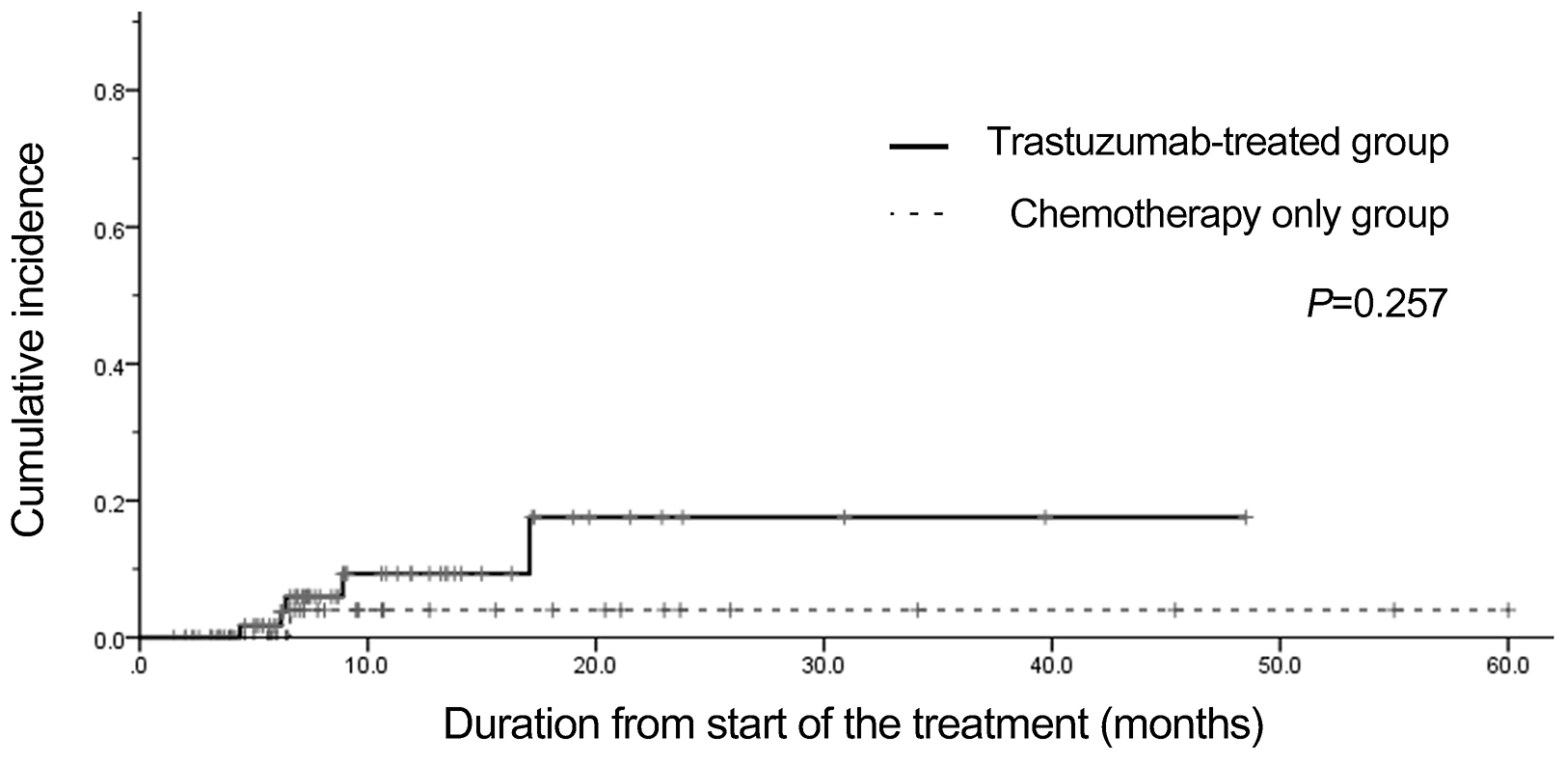
Cumulative incidence of asymptomatic drop in LVEF in both groups

According to the definition described above, the incidence of TIC was 7.1% (5/70). The cumulative incidence of cardiac dysfunction was not different in the trastuzumab-treated group compared with the chemotherapy- only group (HR, 3.47; 95% CI, 0.40–29.8; *P*=0.257; Figure 1). No case occurred between 1 and 3 months from the last dose of trastuzumab.

### Clinical course of the patients with TIC

**Figure 2:**
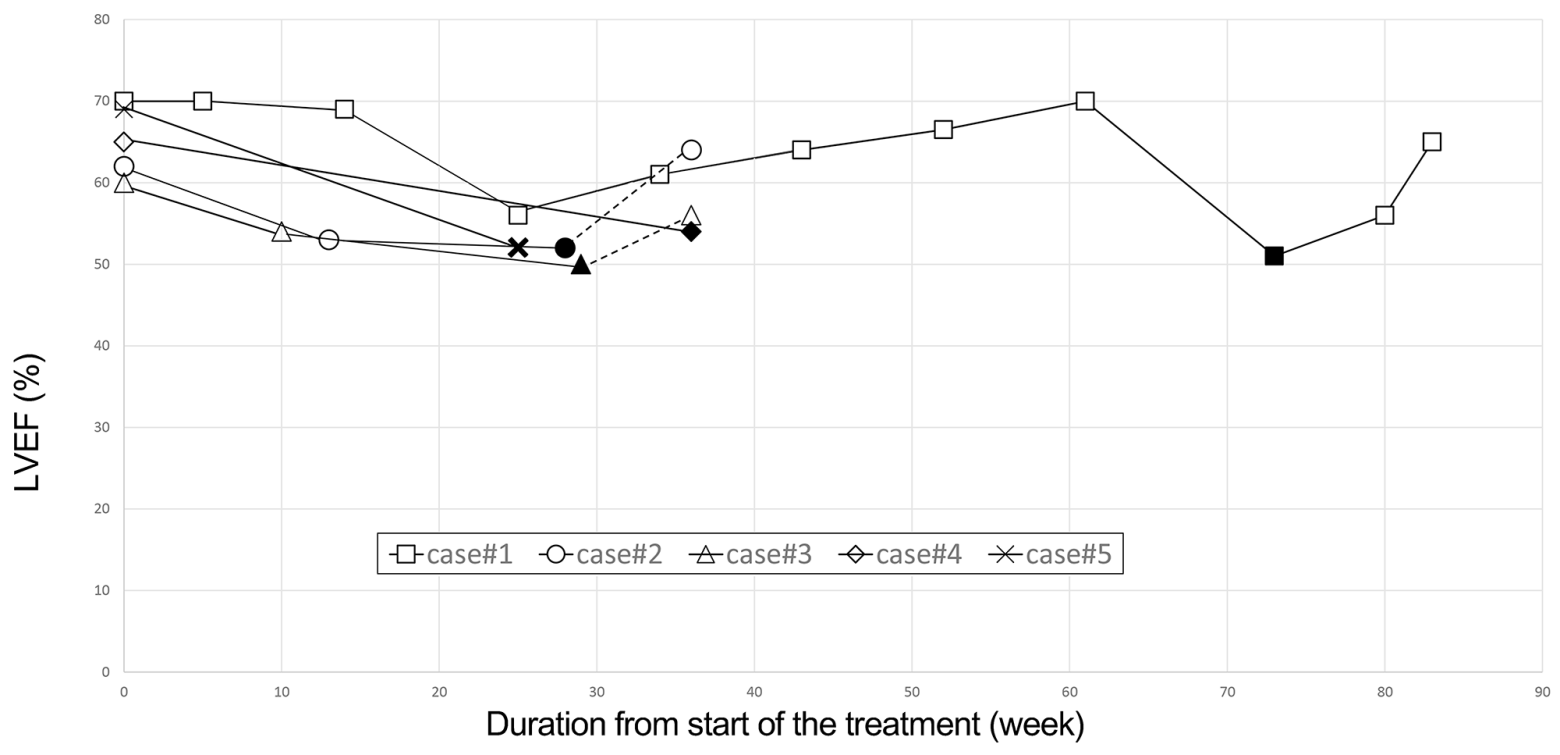
Clinical course of four patients who experienced an asymptomatic drop in LVEF The hollow dots indicate LVEF as the time progressed from the start of treatment. Filled dots indicate the occurrence of a significant drop in LVEF.

There were five patients with TIC (Case#1, Case#2, Case#3, Case#4, and Case#5) (Figure 2).

Case#1 (73-year-old male patient) experienced a significant asymptomatic drop in LVEF of 19 percentage points to 51% at week 73, after the 24th treatment cycle of trastuzumab maintenance followed by capecitabine plus cisplatin combined with trastuzumab. Because the protocol called for treatment to be continued when LVEF ≥ 45%, the patient continued treatment with trastuzumab and LVEF recovered to 65% at week 83. Case#2 (69-year-old male patient) presented with a significant asymptomatic drop in LVEF of 10 percentage points to 50% at week 29 after the 7th cycle of treatment with trastuzumab combined with 5-FU and cisplatin. The patient continued the same dose of trastuzumab plus 5-FU but omitted cisplatin because of general weakness, and LVEF recovered to 56% at week 36. Case#3 (56-year-old male patient) presented with a significant asymptomatic drop in LVEF of 14 percentage points to 53% at week 27 after 8 cycles of trastuzumab treatment combined with capecitabine and cisplatin. The patient stopped the treatment due to cancer progression at week 28. A follow-up cardiac evaluation could not be performed because the patient rapidly progressed and succumbed to cancer. Case#4 (74-year-old female patient) exhibited a significant asymptomatic drop in LVEF from 65% to 54% at week 38 after 9 cycles of trastuzumab monotherapy following 6 cycles of combination therapy with trastuzumab, 5-FU, and cisplatin. The patient changed her regimen due to disease progression, and a follow-up test was not performed. Case#5 (80-year-old male patient) presented with a significant, asymptomatic drop in LVEF of 17 percentage points to 52% at week 26 after 8 cycles of trastuzumab combined with 5-FU and cisplatin. Because the patient succumbed to the cancer, subsequent cardiac evaluation could not be conducted.

### Clinical factors related to TIC

We compared clinical factors according to the occurrence of cardiac dysfunction separately in the trastuzumab-treated group. The median number of cycles of trastuzumab was 10 (range, 8–38) in the patients with cardiac dysfunction, which was not different compared to patients without cardiac dysfunction (median, 8; range, 1–62; *P*=0.403). None of the patients with underlying cardiac disease experienced TIC. Among the clinical factors possibly related to trastuzumab-induced cardiac dysfunction, aging was the only significant factor to affect the occurrence of TIC (Table 2; HR, per age in year, 1.16; 95% CI, 1.02–1.31; *P*=0.019).

**Table 2:** Cox regression models1 for the incidence of TIC2 by risk factors

Variables		Number of Patients	Incidence		HR	95% CI for HR	*P-*Value
			No	%			
			Median (range) for continuous variables				
Age (years)			59 (30–80)		1.16	1.03–1.31	0.019
Gender							
	Male	52	4	7.7	1.49	0.17–13.4	0.722
	Female	18	1	5.6	1		
BMI (kg/m^2^)			21.4 (13.8–32.5)		0.94	0.69–1.28	0.701
Hypertension							
	Yes	18	2	11.1	1.74	0.29–10.5	0.545
	No	52	3	5.8	1		
Diabetes							
	Yes	8	0	0	N/A		N/A
	No	62	5	8.1			
Smoking							
	Current/Former	38	0	0	N/A		N/A
	Never	32	5	15.6			
Baseline LVEF (%)							
	≥55 and ≤60	20	1	5.0	1.60	0.18–14.3	0.677
	≥60	50	4	8.0	1		
Baseline serum CRP (mg/L)			13.3 (0–201)		1.00	0.98–1.02	0.799
Total cholesterol (mg/dL)			153.5 (74–343)		1.00	0.99–1.02	0.769
Cycle (number)			9 (1–62)		0.99	0.92–1.08	0.844
Cumulative dose (mg/kg)			56 (8–374)		1.00	0.98–1.02	0.927
Combined cytotoxic chemotherapy							
	FP	31	4	12.9	6.74	0.74–61.6	0.091
	SP/XP	39	1	2.6	1		

BMI, body mass index; CRP, C-reactive protein; FP, fluorouracil plus cisplatin chemotherapy; HR, hazard ratio; LVEF, left ventricular ejection fraction; SP, TS-1 plus cisplatin chemotherapy; TIC, trastuzumab-induced cardiotoxicity; XP, capecitabine plus cisplatin chemotherapy; ^1^trastuzumab treatment group only; N=70; ^2^TIC was defined as at least one of the following: decreased LVEF ≥10% points to a value of <55%, severe congestive heart failure, or cardiac death.

## DISCUSSION

In the present study, we assessed the incidence of cardiac dysfunction in HER2-positive gastric cancer patients who were treated with cytotoxic chemotherapy in combination with trastuzumab. The incidence of TIC was 7.1%, which was not higher than the incidence in patients treated with chemotherapy only. The only clinical factor possibly related to cardiotoxicity was age greater than 70 years.

Cardiotoxicity is one of the well-known toxicities caused by trastuzumab. In contrast to myocardial injury induced by anthracycline, cardiotoxicity induced by trastuzumab was thought to be generally reversible by the discontinuation of trastuzumab administration and not dose-dependent or functionally related to the contractile elements of the heart [12]. The incidence of cardiotoxicity was approximately 0.4–4.0% in CHF and 4.1–10.4% in left ventricular dysfunction in a large-scale adjuvant trastuzumab breast cancer trial [12]. In a pivotal study reported by Slamon et al., the metastatic breast cancer patients who received the concurrent treatment of trastuzumab and anthracycline had a high incidence of cardiotoxicity that accounted for 27% of symptomatic or asymptomatic cardiac dysfunction [2]. The incidence of TIC was also influenced by exposure to anthracycline [13].

As anti-HER2 therapeutic agents were administered to HER2-positive gastric cancer patients, cardiac safety was also carefully considered, similarly to breast cancer. However, several clinical factors possibly related to cardiotoxicity in breast cancer patients, including obesity [6], previous exposure to anthracycline [7], and radiation to the chest [7], are not frequently found in gastric cancer patients. Additionally, gastric cancer patients include a greater number of males, the elderly, and members of the Asian population, compared to breast cancer patients. Therefore, the cardiotoxicity profiles of HER2-positive gastric cancer patients treated with trastuzumab are likely different from breast cancer patients.

Despite its rarity in the clinical trials conducted thus far in the patients with HER2-positive gastric cancer, the cardiotoxicity induced by trastuzumab was infrequent, reversible, and not severe, similar to observations in breast cancer. The rate of all cardiac adverse events, grade 3 or 4 cardiac adverse events and a significant drop in LVEF to an absolute value <50% were 6%, 1%, and 5%, respectively, in the trastuzumab and chemotherapy combination group in the ToGA trial [11]. A phase II clinical trial of trastuzumab, combined with TS-1 and cisplatin, reported that half of the patients (15/30) experienced a decline in LVEF from baseline during the treatment, and six patients (20%) presented with a >10% drop in LVEF from baseline values [14]. In addition, a retrospective study indicated that 26% of the HER2-positive metastatic esophagogastric cancer patients treated with trastuzumab experienced asymptomatic cardiotoxicity (defined as a drop of more than 10% in LVEF in the third-month echocardiogram) and 8.6% experienced symptomatic cardiotoxicity [15] (Supplementary Table 1).

In the present study, during a median 9.3 month (range, 3.5–68.2) follow-up period, we found a significant asymptomatic drop in LVEF in 7.1% of the gastric cancer patients treated with a trastuzumab combination. There was no symptomatic cardiac dysfunction related to trastuzumab. Considering that the median progression-free survival time of the patients who were treated with a trastuzumab combination therapy in the ToGA trial was 6.7 months, the patients with inoperable or metastatic HER2-positive gastric cancer were expected to receive trastuzumab for approximately 6–7 months. In this study, the rough incidence of a significant asymptomatic drop in LVEF at 7 months was 5.1% in the trastuzumab-treated group and 2.7% in chemotherapy-only group (*P*=0.227). Most of the significant, asymptomatic drops in LVEF were reversible within 6 weeks of the discontinuation of trastuzumab, or within 8 weeks despite the continuation of trastuzumab. Therefore, we believed that the differential diagnosis of another cardiac disease was required for the patients with persistently decreased LVEF.

Although 78 deaths occurred, none were related to TIC, and one patient experienced stable angina pectoris 8 months after the discontinuation of trastuzumab. In addition, either overall survival (HR, 0.936; 95% CI, 0.290–3.023; *P*=0.912) or progression free survival (HR, 0.875; 95% CI, 0.350–2.186; *P*=0.775) was different in the patients with TIC compared with those without TIC.

The analysis of clinical factors related to cardiac dysfunction revealed that old age was a possible risk factor for TIC in inoperable or metastatic HER2-positive gastric cancer patients (Table 2). Other possible risk factors, including baseline LVEF lower than 60%, chronic diseases, and smoking history were not statistically significant. As well, although we analyzed the correlation between serum C-reactive protein (CRP), a classic biomarker for heart failure [16], and total cholesterol, another classic biomarker for cardiovascular disease [17], we did not detect a significant association (Table 2). However, these results are very preliminary due to the small number of cases.

The major limitations of our study were its small number of patients, the introduction of a potential selection bias because not all of the patients were included in the analyses, and heterogeneous therapeutic regimens. Due to a lack in the number of events, the evaluation of several clinical factors that might have been related to cardiotoxicity was insufficient. Moreover, only 33 patients had a baseline and follow-up results evaluated by echocardiogram and several parameters for diastolic dysfunction could not be fully analyzed. As well, other cardiac markers, including high-sensitivity C-reactive protein (hs-CRP) and B-type natriuretic peptide (BNP), were also not evaluated. Despite these limitations, the significance of this report included showing the frequency and detailed characteristics of cardiac dysfunction caused by trastuzumab in gastric cancer patients from prospectively-collected databases.

It was generally accepted that TIC was infrequent and reversible. However, a lack of knowledge regarding TIC in patients with gastric cancer remains, and we are not yet able to predict the long-term cardiac consequences of trastuzumab [18]. Awareness and continuous observation of cardiac events in HER2-positive gastric cancer patients receiving anti-HER2 treatment will be needed in clinical practice, especially in elderly patients.

## METHODS

### Study population

Patients who were diagnosed with HER2-positive gastric cancer between December 2005 and April 2015 at the Yonsei Cancer Center, Seoul, Republic of Korea were included in this study. The criteria for eligibility were as follows: 1) histologically proven HER2-positive, advanced gastric cancer; 2) age ≥ 18 years; 3) inoperable locally advanced, recurrent, or metastatic disease; 4) an Eastern Cooperative Oncology Group (ECOG) performance status of 0–2; 5) adequate cardiac function [LVEF ≥ 55% measured by echocardiography or a multiple-gated acquisition (MUGA) scan]; and 6) at least one follow-up echocardiography or MUGA scan test during the first-line treatment (whether or not the regimen included anti-HER2 treatment).

The exclusion criteria were as follows: 1) operable gastric cancer; 2) the presence of other active malignancies; 3) treatment with other anti-HER2 target agents including lapatinib or trastuzumab emtansine; 4) clinically severe cardiac problems [including documented CHF, uncontrolled coronary artery disease (angina pectoris requiring antianginal medication or transmural infarction on electrocardiography (ECG)], uncontrolled hypertension (systolic blood pressure > 180 mmHg or diastolic > 100 mmHg), high risk arrhythmias, or significant valvular disease; or 5) the presence of other severe medical illnesses.

HER2 positivity was defined using the HER2 scoring criteria for gastric cancers by Hofmann et al. [19] and was confirmed using immunohistochemical staining (IHC; Hercep Test, Dako, Denmark) or silver *in situ* hybridization (SISH; Ventana Discovery XT system, Ventana/Roche, United States) by experienced pathologists at the Yonsei Cancer Center.

Clinical information, including gender, age, body mass index (BMI), baseline LVEF, underlying chronic diseases, and smoking history, was reviewed and documented.

The ethical principles for medical research established by the World Medical Association Declaration of Helsinki were followed throughout the study. The Severance Hospital, Seoul, Korea Institutional Review Board reviewed and approved this study (IRB approval number: 2014-2763-001)

### Treatment

This study included HER2-positive advanced or metastatic gastric cancer patients who received chemotherapy with or without trastuzumab not only in clinical practice and clinical trials. The patients were treated with one of the following first-line treatment regimens: trastuzumab treatment regimens including trastuzumab in combination with fluorouracil (5-FU) plus cisplatin, trastuzumab in combination with capecitabine plus cisplatin [11], or trastuzumab in combination with S-1 plus cisplatin [14]. All of the therapeutic regimens that included trastuzumab involved the intravenous infusion of trastuzumab at a dose of 8 mg/kg on day 1 of the first cycle, followed by 6 mg/kg every 3 weeks until disease progression. Chemotherapeutic regimens without anti-HER2-targeted therapy included 5-FU plus cisplatin, capecitabine plus cisplatin, or TS-1 plus cisplatin. The regimens used for the patients in this study were the same as those previously described [11, 20, 21].

### Cardiac monitoring

The performance of physical examinations, an ECG, and an assessment of LVEF by echocardiography or MUGA scanning were planned at baseline and every 9–12 weeks during first line treatment. An additional cardiologic workup was conducted when the patients complained of cardiologic symptoms including chest pain, chest discomfort, or dyspnea on exertion.

### Definitions

In this study, cardiac dysfunction included a significant drop in LVEF, severe CHF, and cardiac death. A significant drop in LVEF was defined as an absolute decline of at least 10 percentage points from the baseline to a value less than 55% identified by a MUGA scan or echocardiogram regardless of symptoms, which were defined in a previous study [8]. Severe CHF was defined as class III or IV according to the New York Heart Association (NYHA) with a significant drop in LVEF [22]. Cardiac death was defined as death that was definitively due to heart failure, myocardial infarction, or primary arrhythmia. Cardiac dysfunction related to treatment with trastuzumab was defined as trastuzumab-induced cardiotoxicity (TIC). Abnormal results were analyzed for up to three months from the date of the last administration of trastuzumab. The definition of reversibility was in accord with an expert consensus suggested by the American Society of Echocardiography [23].

### Statistical analysis

The clinical differences between the groups that received first-line chemotherapy with or without HER2-targeted therapy were analyzed using the Chi-square, Fisher’s exact, and Mann-Whitney U tests. Cumulative incidences of cardiac dysfunction were compared using a Kaplan-Meier analysis. The incidence of cardiac dysfunction between each of the groups with different clinicopathological features was compared using logistic regression models. A *P* <0.05 was considered statistically significant. All statistical analyses were performed using IBM SPSS version 23.0 for Windows (IBM, Armonk, NY, USA).

## SUPPLEMENTARY MATERIALS TABLE


