# Supplementary material for: Cardiotoxicity of trastuzumab in patients with HER2-positive gastric cancer
Source: Oncotarget. 2017 Jun 27;8(37):61837–45. doi: 10.18632/oncotarget.18700 (PMC5617468; doi:10.18632/oncotarget.18700)
Supplement: Supplementary file 1 [file oncotarget-08-61837-s001.pdf]

Cardiotoxicity of trastuzumab in patients with HER2-positive gastric cancer

SUPPLEMENTARY MATERIALS

Supplementary Table 1: Comparison of TIC in four studies regarding trastuzumab combination treatment in gastric cancer patients

Combination regimen	Total number of the patientsin TG	Inclusion criteria of baseline LVEF	Definition of TIC	Reported incidence of TIC (%)	Reported incidence of any grade of drop in LVEF (%)	Reversibility reported	Reference
Trastuzumab + FP or XP	298	≥50%	≥10% drop in LVEF to an absolute value <50%	11/237 (5%)	N/A	N/A	[11]
Trastuzumab+ SP	30	≥50%	>10% drop in LVEF from baseline	6/30(20%)	15/30(50%)	N/A	[14]
Trastuzumab + FP or XP	23	N/A	>10% drop in LVEF from baseline	6/23(26%)	N/A	N/A	[15]
Trastuzumab + FP or XP or SP	70	≥55%	an absolute decline of at least 10 % points from the baseline to a value <55%	5/70(7.1%)	44/70(62.9%)	Yes*	Present study§

TIC, trastuzumab-induced cardiotoxicity; FP, 5-fluorouracil plus cisplatin; N/A, not available; SP, S-1 plus cisplatin; TG, trastuzumab-treated group; XP, capecitabine plus cisplatin; *In most cases, a significant asymptomatic drop in LVEF was reversible within 6 weeks by discontinuation of trastuzumab, or in 8 to 10 weeks despite continuation of trastuzumab, except in one case in which it was impossible to re-assess due to the cancer-related death of the patient; §The present study included a portion of the patients in the ToGA trial [11] and a phase II study of trastuzumab in combination with S-1 and cisplatin [14]
